# Occurrence and Exposure Assessment of Major Mycotoxins in Foodstuffs from Algeria

**DOI:** 10.3390/toxins15070449

**Published:** 2023-07-06

**Authors:** Azem Belasli, Marta Herrera, Agustín Ariño, Djamel Djenane

**Affiliations:** 1 Food Quality and Safety Research Laboratory, Department of Food Sciences, Mouloud Mammeri University, P.O. Box 17, Tizi-Ouzou 15000, Algeria; azem_belasli@live.fr; 2 Facultad de Veterinaria, Instituto Agroalimentario de Aragón-IA2, Universidad de Zaragoza-CITA, 50013 Zaragoza, Spain; aarino@unizar.es

**Keywords:** mycotoxins, aflatoxins, ochratoxin A, deoxynivalenol, traditional foods, Algeria

## Abstract

Cereal-based products, nuts and dried fruits are staple foods for the Algerian population. However, these foodstuffs may be sources of harmful mycotoxins, with negative impacts on human health. The purpose of this study was to investigate the occurrences and levels of aflatoxins (B1, B2, G1 and G2), ochratoxin A (OTA), deoxynivalenol (DON) and zearalenone (ZEA) in 198 samples of nuts, dried fruits and cereal products commercialized in Algeria, as well as to calculate the estimated daily intakes (EDIs). Aflatoxins were found in 26.2% of the nut samples (in peanuts and almonds, but not in walnuts), 38.7% of the dried fruit samples (in dried figs, dates and *bradj* pastries) and 47.9% of the cereal-based products (in *rechta* noodles and *metlou* bread, but not in couscous), with mean concentrations ranging from 0.03 to 0.49 μg/kg. OTA occurred in 16.9% of the cereal-based samples, averaging 0.15 μg/kg, but it was not detected in nuts or dried fruits. The incidence of DON in the cereal-based products was 85.9% on average, with a mean concentration from 90 to 123 μg/kg. ZEA mycotoxin was not detected in any samples. Four peanut samples exceeded the EU maximum level for aflatoxin B1 set at 2 μg/kg, while three of them surpassed the maximum level for the sum of aflatoxins (4 μg/kg). Traditional foods such as *bradj*, *rechta* and *metlou* were significant sources of aflatoxins, with MOE (margin of exposure) values ranging from 648 to 9333, indicating a potential risk for the Algerian population.

## 1. Introduction

Mycotoxins are considered to be among the most significant food contaminants in terms of their negative impact on public health [1]. They are produced by toxigenic fungi in the field and/or during storage, and their levels can vary depending on the season, growing area and storage conditions. Additionally, they are very stable in different circumstances and thus difficult to remove from the food chain. This causes worldwide losses of crop production, affecting feed and food safety, food security and international trade [2].

The mycotoxin-producing fungi are mainly species of the *Fusarium*, *Aspergillus* and *Penicillium* genera, and more than 400 types of mycotoxins have been described [3]. Some of the mycotoxins of the greatest public health and agroeconomic significance include aflatoxins (AFs), ochratoxin A (OTA), deoxynivalenol (DON) and zearalenone (ZEA). They can cause a wide range of toxicological effects both on human and animal health, ranging from the development of carcinogenic, teratogenic and mutagenic effects to the hormonal and immunosuppressive disorders that result from consuming mycotoxin-contaminated food or feeds [4].

During the last decade, a small number of studies have been published on the incidence of mycotoxins in food products from North African countries. Cereal products, dried fruits and nuts are susceptible to mold growth and mycotoxin formation before or during harvest and storage under critical environmental conditions. Cereal-based products are staple foods in the Mediterranean regions of North Africa, where consumption in the form of couscous, pasta and traditional bread is a cultural tradition [5]. Nowadays, dried fruit and nut consumption is also widespread in African countries, and they are very appreciated and much used in Algeria. In fact, many traditional meals and cakes highly consumed in this country are made with dates, dried figs, almonds and peanuts [6].

Algeria, a North African country bordered to the north by the Mediterranean Sea, has a mostly hot and dry climate, but with some humid areas, which favor the growth of molds and the production of mycotoxins [7]. However, there are not enough studies available on the incidence of mycotoxins in food, nor on the exposure of the population to these contaminants or health risk assessment. This situation, combined with the absence of regulations on the maximum limits for mycotoxins in raw materials and foodstuffs, makes prevention and control difficult. Indeed, food safety issues pose major challenges due to several reasons, such as the use of low-quality agricultural inputs, the neglect of good pre- and post-harvest farming practices and poor management of food handling and processing. There is also a lack of incentive strategies for, education on and awareness of mycotoxins [8].

Currently, only aflatoxins are regulated in Algeria and most African countries, despite the existence of other mycotoxins in the region. The aflatoxin B1 (AFB1) and total aflatoxin (AFs: sum of AFB1, AFB2, AFG1 and AFG2) regulatory limits have been set in peanuts, nuts and cereals at 10 and 20 μg/kg, respectively [9]. Even in most cases, the existing AF regulations are only considered for commodities with trade values leaving the local population with unsafe agricultural products. Up until now, Algeria has not set maximum levels for OTA, DON or ZEA in foodstuffs.

Therefore, the establishment of preventive strategies for mycotoxin contamination is necessary to mitigate health risks. This action plan includes good agricultural and storage practices, the regulation of maximum levels, contamination control and exposure assessment. Studies on mycotoxin exposure estimation contribute significantly to risk assessment and management, as well as to the establishment of legal regulations for the monitoring and control of mycotoxins in the food chain.

In fact, for the regulated mycotoxins, tolerable dietary intakes (TDIs) or provisional maximum tolerable dietary intakes (PMTDIs) have been established by the Joint Expert Committee on Food Additives (JECFA). As AFs are carcinogenic to humans (IARC Group 1), there is no safe intake level; thus, it is recommended that their levels in food be as low as reasonably possible (ALARA) [10]. For AFs, the Scientific Committee of the EFSA (European Food Safety Authority) therefore recommends using a different approach, known as the margin of exposure (MOE). An MOE value of 10,000 (ten thousand) or higher would be of low concern from a public health point of view [11]. Similarly, OTA is a well-known nephrotoxic compound that is potentially carcinogenic to humans (Group 2B), and the EFSA recently suggested applying a margin-of-exposure (MOE) approach to characterize its risk [12]. The IARC has classified DON and ZEA in Group 3 (not classifiable as to its carcinogenicity to humans). For these mycotoxins, TDIs have been established at 1 and 0.25 μg/kg bw/day, respectively [13,14].

To the best of our knowledge, very few studies have been carried out on the mycotoxin occurrence and exposure assessment of major mycotoxins in nuts, dried fruits and traditional cereal-based products in Algeria. Some researchers have been interested in the contamination of nuts and dried fruits by AFs, providing occurrence data on pistachios, almonds, peanuts, walnuts and dried figs [6,15]. There is only one report on the multi-mycotoxin occurrence and exposure assessment approach in foodstuffs from Algeria [16]; so far, the available information has been rather scarce.

Considering this fact, the aim of the present study was to determine the occurrences and levels of major mycotoxins (aflatoxins, ochratoxin A, deoxynivalenol and zearalenone) in susceptible food commodities available in Algerian markets. Subsequently, mycotoxin analyses of dried fruits, nuts and traditional cereal-based products were considered to estimate the potential contribution to the dietary exposure of Algerian consumers.

## 2. Materials and Methods

### 2.1. Study Area and Sample Collection

**Figure 1:**
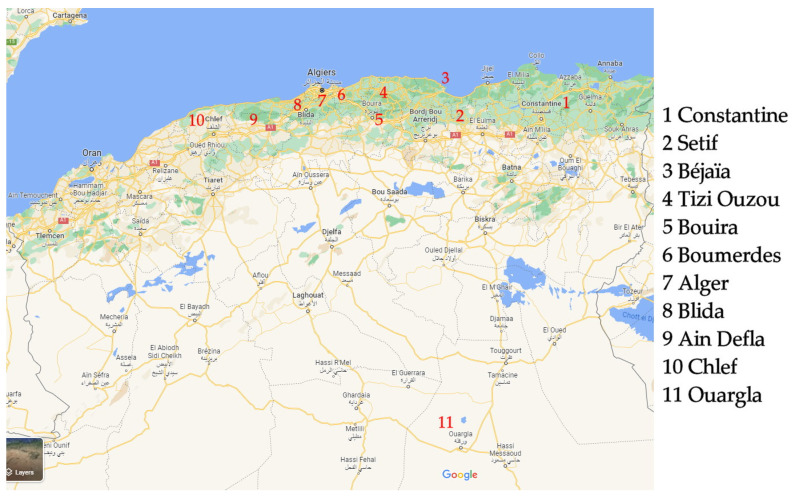
Geographical distribution of food samples from Algeria (adapted from Google Maps 2023 available at https://www.google.com/maps (accessed on 31 May 2023); Map data 2023 Inst. Geogr. Nacional, Google).

A total of 198 samples were randomly purchased from different markets, hypermarkets and food retailers in eleven administrative territories in Algeria (Constantine; Setif; Béjaïa; Tizi Ouzou; Bouira; Boumerdes; Alger; Blida; Ain Defla; Chlef; and Ouargla) during 2019 (Figure 1). 

The samples were grouped as nuts (n = 65), dried fruits (n = 62) and cereal-based products (n = 71). The sampling of nuts consisted of peanuts (n = 24), almonds (n = 21) and walnuts (n = 20). The dried fruits included dried figs (n = 29), dates (n = 20) and a traditional date-filled confectionery called *bradj* (n = 13). Cereal-based products comprised couscous (n = 27), *rechta* noodles (n = 26) and Algerian *metlou* bread (n = 18). The dried fruits and nuts were sold in bulk at local markets, and no labels were available to verify their origin or shelf life. According to information provided by vendors, most samples of dried figs and dates came from Algeria, while peanuts, almonds and walnuts were imported. Couscous was made from wheat produced in Algeria, and most samples were purchased in packages with a shelf life of 24 months. Most of the traditional food samples (*bradj*, *metlou* and *rechta*) were handmade or homemade from local ingredients and sold without shelf-life indication.

All samples were homogenized using a blender, and then a 200 g subsample was collected in a polyethylene container and kept at −20 °C until mycotoxin analysis.

**Figure 2:**
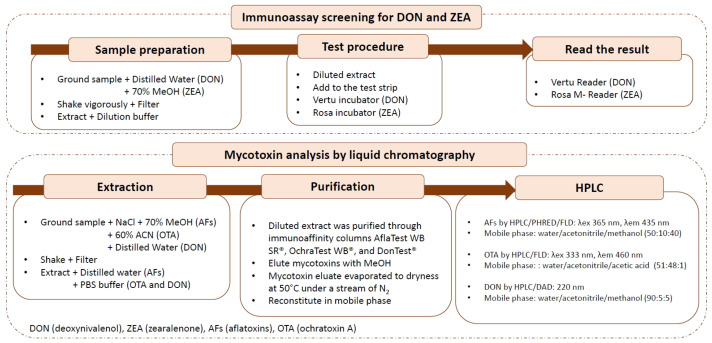
Schematic diagram of the analytical methodology for mycotoxins in the samples.

Figure 2 presents a schematic diagram of the analytical methodology that is described in Section 2.2, Section 2.3 and Section 2.5.

### 2.2. Preliminary Screening Procedure for Deoxynivalenol and Zearalenone

The analyses of DON and zearalenone in cereal products (couscous, *rechta* and *metlou*) were previously carried out with quantitative lateral-flow immunoassays, according to the instructions of the manufacturers. For deoxynivalenol, DON-V lateral-flow strips (P/N: 176002072), consumables and the Vertu quantitative strip reader were obtained from Vicam (Milford, MA, USA). Zearalenone was determined using the Rapid One Step Assay (ROSA) quantitative test kits from Charm Sciences (Lawrence, MA, USA) using the ROSA FAST 5 test strip (P/N: ZEARQ-FAST5), read in the ROSA-M Reader. Both test procedures are recognized by the FGIS Official Mycotoxin Testing Services belonging to the USDA (United States Department of Agriculture) [17]. The immunoassay methods were validated in-house with respect to recovery and precision using couscous samples spiked at 1000 μg DON/kg and 150 μg ZEA/kg using stock solutions of mycotoxins supplied by Sigma-Aldrich (St. Louis, MO, USA). The recoveries for DON and ZEA were 95% and 99%, respectively, with repeatability (RSDr) lower than 20%. In order to verify that these assays were fit for the purpose, positive samples for DON were also subsequently analyzed via high-performance liquid chromatography (HPLC). As the ZEA mycotoxin was not detected in any samples, this mycotoxin was not investigated further via HPLC.

### 2.3. Chemicals and Reagents for Chromatographic Analysis

Reagents sodium chloride (NaCl) and phosphate-buffered solution (PBS) (with disodium hydrogen phosphate (10 mM), potassium chloride (2.7 mM) and sodium chloride (140 mM)) at pH 7.4 were supplied by Panreac (Barcelona, Spain). The immunoaffinity columns (IACs) AflaTest WB SR^®^, OchraTest WB^®^ and DonTest^®^ (VICAM, Watertown, MA, USA) were used to purify the extracts. Nitrogen C55 for solvent evaporation was purchased from Carburos Metálicos (Barcelona, Spain). HPLC-grade acetonitrile, ethyl acetate and methanol were provided by Scharlau (Scharlab, Barcelona, Spain), while ultrapure water was obtained from a Milli-Q water purification system (18.2 MU cm^−1^, Millipore, Bedford, MA, USA).

The stock solutions of aflatoxins (B1, B2, G1 and G2), ochratoxin A and deoxynivalenol were provided by Sigma-Aldrich (St. Louis, MO, USA). AFs consisted of a mix with 1 μg AFB1 and AFG1 and 0.3 μg AFB2 and AFG2 in methanol. An intermediate solution for AFs was made of the original mix, and working standard solutions were prepared at different concentrations between 0.02 and 5 ng/mL for AFB1, G1, B2 and G2 in the mobile phase consisting of water/acetonitrile/methanol (50:10:40). OTA and DON stock solutions were composed of 10 μg OTA/mL in acetonitrile and 200 μg DON/mL in ethyl acetate/methanol (95:5), respectively, and their intermediate solutions were made with methanol. OTA working standards were prepared at 0.25–3 ng/mL in the mobile phase consisting of water/acetonitrile/acetic acid (51/48/1), whereas DON working standards were prepared at 30–500 ng/mL in the mobile phase made up with water/acetonitrile/methanol (90:5:5). Stock and intermediate solutions were stored at −20 °C, while working calibration solutions were stored at 4 °C and renewed every week. The calibration curves for the analyzed mycotoxins are shown in Supplementary Figures S1 and S2.

The researchers regularly used certified reference materials (CRMs) and participated in proficiency tests. With each batch of analytical samples, certified reference materials purchased from Biopure (Romer Labs, Tulln, Austria) were analyzed to check the ongoing precision and recovery. Thus, maize flour containing aflatoxins (AFB1 at 7.3 ± 2.4 μg/kg; AFB2, AFG1 and AFG2 at <1 μg/kg each), OTA at 9.4 μg/kg and DON at 821 ± 64 μg/kg were used. The performance values obtained were within the acceptable margins outlined in Commission Regulation (EC) No. 401/2006 [18].

Considering safety notes, all used laboratory glassware was treated with an aqueous solution of sodium hypochlorite (5%) before discarding to minimize health risks regarding mycotoxin contamination [19].

### 2.4. HPLC Equipment and Chromatographic Conditions

The LC system consisted of an Agilent Technologies 1100 high-performance liquid chromatograph coupled to diode-array (DAD) and fluorescence (FLD) detectors (Agilent Technologies, Santa Clara, CA, USA). Separation was carried out on an LC column Ace 5 C18, 250 mm × 4.6 mm, 5 μm particle size (Análisis Vínicos, Ciudad-Real, Spain), at 50 °C for AFs and 25 °C for OTA and DON. A manual injector system equipped with a 100 μL injector loop was used. The isocratic mobile phases for AFs (water/acetonitrile/methanol: 50:10:40), DON (water/acetonitrile/methanol: 90:5:5) and OTA (water/acetonitrile/acetic acid: 51/48/1) were pumped at flow rates of 0.7 mL/min for aflatoxins and 1.0 mL/min for OTA and DON. The FLD detector was set at 365 nm (excitation) and 435 nm (emission) for aflatoxins, and at 333/460 nm for OTA, whereas the diode-array detector (DAD) for DON was set at 220 nm. The LC system was connected to a photochemical reactor for enhanced detection (PHRED detector) (LCTech UVE, Dorfen, Germany) for the quantification of aflatoxins via post-column photochemical derivatization. The LCTech UVE was connected between the column and fluorescence detector and was set at 254 nm.

### 2.5. Mycotoxin Analysis by HPLC

The analytical methods were performed according to CEN standards and manufacturer recommendations, with slight modifications. Accordingly, they were previously optimized and validated in-house in compliance with the guidelines indicated in Commission Regulation No. 401/2006 for methods of sampling and analysis for the official control of mycotoxins [18].

The procedure for the extraction, cleanup and determination of total aflatoxins (AFB1, AFB2, AFG1 and AFG2) was based on the method EN ISO 16050:2011 [20]. A representative ground sample of 5 g was extracted with 1 g sodium chloride and 25 mL methanol/water (7:3, *v*/*v*) using an ultraturrax homogenizer (IKA Labortechnik M20, Staufen, Germany) for 2 min. The extract was filtered through Whatman No. 4 filter paper (Symta, Madrid, Spain), and 15 mL was diluted with 30 mL of distilled water and purified through the immunoaffinity column AflaTest WB SR^®^, according to the instructions of the manufacturer. Subsequently, aflatoxins in the extract bound to the antibody, impurities were removed with water and then the mycotoxin was desorbed with methanol. The collected methanolic eluate with aflatoxins was filtered through a glass-fiber filter (Symta, Madrid, Spain) and evaporated to dryness at 50 °C under a stream of nitrogen in a sample concentrator (Stuart instruments, Cambridge, UK). The dried eluate was reconstituted with 1 mL of mobile phase and filtered through a 0.45 μm filter (Análisis Vínicos, Ciudad-Real, Spain). The samples were injected twice (duplicate injection) into the LC-PHRED-FLD system. The limit of detection (LD) was 0.02 μg/kg for each of the aflatoxins: B1, B2, G1 and G2.

In short, the method EN 14132:2009 for ochratoxin A [21] starts with 5 g of sample vortexed with 20 mL of acetonitrile/water (6:4, *v*/*v*) for 2 min and filtered through Whatman No. 4 filter paper. The volume of 10 mL was diluted with 25 mL of PBS and filtered through a glass-fiber filter. An amount of 14 mL of the diluted filtered extract was completely passed through the immunoaffinity column OchraTest WB^®^, following the instructions of the supplier. The collected eluate was evaporated with nitrogen, and aliquots of 100 μL of the ochratoxin-containing eluate were injected twice into the liquid chromatograph coupled to an FLD detector. The limit of detection (LD) of OTA was 0.10 μg/kg.

The method EN 15891:2010 for deoxynivalenol [22] starts by mixing 5 g of sample with 20 mL of distilled water for 2 min and filtering through Whatman No. 4 filter paper. An amount of 4 mL of the extract was diluted with 16 mL of PBS and passed through a DonTest^®^ IAC. The IAC was then eluted with 2 mL of methanol, and the solvent was evaporated to dryness at 50 °C under a gentle stream of nitrogen. Finally, the eluate was dissolved in 500 μL of a mixture of water/acetonitrile/methanol (90/5/5), filtered through a 0.45 μm filter and 100 μL was injected twice into the LC-DAD system. The limit of detection (LD) of DON was 33 μg/kg.

### 2.6. Data Analysis

The results from the mycotoxin analyses were performed using the software package OpenLab CDS (Agilent Technologies, Santa Clara, CA, USA). A sample was considered positive for a tested mycotoxin when the concentration was above the limit of detection (LD). Samples below the LD (non-detects or left-censored data) were assigned a value of one-half the LD for the interpretation of the results and exposure assessment purposes [23]. The descriptive analysis of the mean, standard deviation (SD) and relative standard deviation (RSD%) was performed with Statistical Package SPSS v21 (IBM Corporation, Armonk, NY, USA).

## 3. Results and Discussion

### 3.1. Mycotoxin Occurrence Data

The analytical methods used were satisfactorily validated for recovery, repeatability and reproducibility in accordance with Commission Regulation (EC) No. 401/2006 [18] and the guidance document SANTE/11312/2021 [24]. The recovery percentages were in the range of from 70 to 120%, and the RSD value for repeatability was less than 20%.

**Figure 3:**
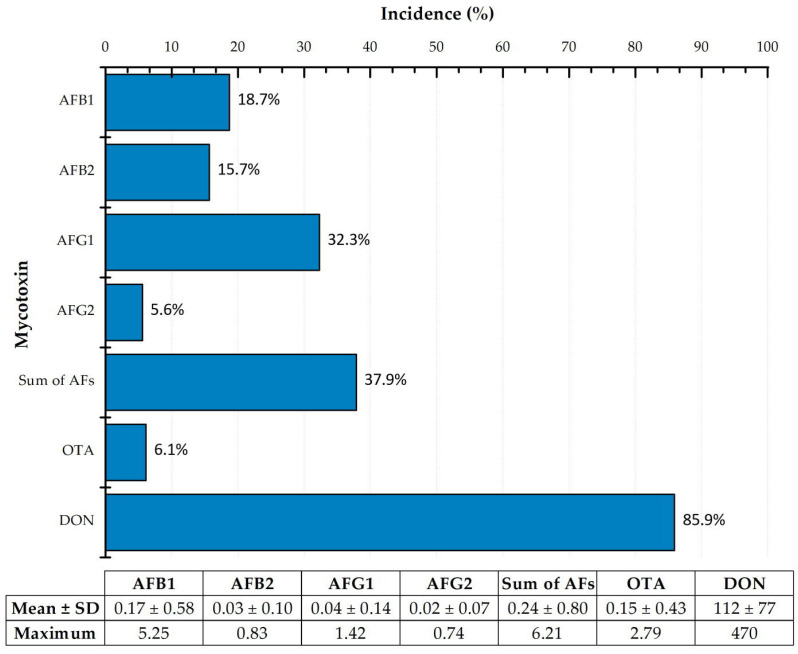
Incidence (%) and contamination levels (μg/kg) of mycotoxins in the 198 food samples.

A total of 198 Algerian food commodities consisting of nuts, dried fruits and cereal-based products were analyzed and evaluated. Figure 3 shows the incidence (% positive), mean concentration (±standard deviation (SD)) and maximum values of the mycotoxins AFs (AFB1, AFB2, AFG1, AFG2), OTA and DON in the different samples. A total of 107 out of 198 samples (54.0%) were contaminated with at least one mycotoxin at variable levels. From the total samples, 75 (37.9%) were contaminated with aflatoxins at levels ranging from 0.21 to 6.21 μg/kg, corresponding to the sum of aflatoxins (AFs). AFB1 occurred in 37 samples (18.7%), and AFG1 in 64 (32.3%), whereas AFB2 and G2 were present in 31 (15.7%) and 11 (5.6%) food samples, respectively. It is remarkable that AFB1 and AFB2 co-occurred in 31 (15.7%) samples, and B1, B2 and G1 in 24 samples (12.1%). OTA occurred in 16.9% of the cereal-based samples (couscous, *rechta* and *metlou*), averaging 0.15 μg/kg, but it was not detected in nuts or dried fruits. The incidence of DON in cereal-based products ranged from 77.8% in couscous to 83.3% in *metlou* and 96.2% in *rechta*, with a mean concentration from 90 to 123 μg/kg. ZEA mycotoxin was not detected in any samples after the analysis via the immunoassay method (LD of 15 μg/kg).

Sample LC chromatograms of positive samples for the analyzed mycotoxins are shown in Supplementary Figures S3–S8.

#### 3.1.1. Occurrences of Aflatoxins and Ochratoxin A in Nuts

In the nut group, AF mycotoxins were detected in 17 out of 65 samples, but none of them contained OTA. Data regarding aflatoxin contamination (AFB1, AFB2, AFG1, AFG2 and the sum of the four aflatoxins) in the 65 nut samples are summarized in Table 1. A total of 17 samples out of 65 (26.2%), corresponding to peanuts and almonds, were contaminated with AFs. However, there was no finding of aflatoxins in walnuts. All positive samples of nuts contained both AFB1 and AFB2, while the prevalence of AFG1 was 16.9%, and that of AFG2 was 10.8%. In the nut group, the highest incidence of AFs was detected in peanuts, with the presence of AFB1 and AFB2 standing out, as they were present in 62.5% of the samples, with mean concentrations of 0.92 μg/kg and 0.16 μg/kg, respectively. The incidence of AFG1 was 45.8% (mean: 0.17 μg/kg), and that of AFG2 was 29.2% (mean: 0.08 μg/kg). AFB1 and AFB2 were detected in 9.52% of the almond samples at a 0.01 μg/kg mean concentration, while AFG1 and AFG2 were not present in almonds. Four peanut samples exceeded the EU maximum level for AFB1 set at 2 μg/kg, while three of them surpassed the maximum content for the sum of aflatoxins (4 μg/kg) [25]. However, aflatoxins were below the maximum contents established in Algerian regulation at 10 μg/kg for AFB1 and 20 μg/kg for the sum of AFs [8,9].

The prevalence of AFB1 and AFB2 in nuts was in accordance with other studies in which both AFs are reported as the most common mycotoxins in peanuts, compared to AFG1 and AFG2, due to *A. flavus* contamination [26]. Aflatoxin G was also less frequently detected and determined at lower levels than AFB1 in nut samples in other studies [27,28].

It is well known that peanuts are a product that is highly susceptible to aflatoxin contamination. The present results of the aflatoxin incidence and concentration are in line with two previous studies carried out in Algeria. Riba et al. [15] reported an incidence of AFs in peanuts of 100%. Ait Mimoune et al. [6] detected aflatoxins in 57% of peanut samples, with 8% of the samples exceeding the Algerian limit for AFB1 (10 μg/kg), while, in almonds, 56% of the samples were positive for AFs. A remarkable incidence of aflatoxins in peanuts has also been reported in other parts of the world: 55% in Zambia [27], 53% in Ghana [29] and 70% in Pakistan [30].

Practical solutions to reduce AFs in food include educational interventions, the application of sorting and cleaning methods for grains and other agricultural products as well as the optimization of processing and control of storage conditions. Several collaborative efforts are currently underway to mitigate mycotoxin exposure on the African continent; for example, the “aflasafe” project (www.aflasafe.com; accessed on 31 May 2023), which uses a biological control technique for the prevention of aflatoxins in maize and peanuts. We also highlight the program for aflatoxin control in Africa (www.aflatoxinpartnership.org; accessed on 31 May 2023), the mission of which is to support agricultural development, safeguard consumer health and facilitate trade by catalyzing, coordinating and increasing effective aflatoxin control along agricultural value chains in Africa.

#### 3.1.2. Occurrences of Aflatoxins and Ochratoxin A in Dried Fruits

Algeria is one of the most important producers of dates and figs, ranking fourth worldwide [31]. In the dried fruit group, AFs were detected in 19 out of 62 samples, but none of them contained OTA. The contamination with AFs in dried figs, dates and a date-filled confectionery called *bradj* is shown in Table 2. Traditional *bradj* contained aflatoxins in 100% of the samples, followed by dried figs (34.5%) and dates (5%). The pattern of contamination was somewhat different from that found in peanuts, as AFG1 was more prevalent (32.6% incidence) than AFB1 (30.6%) and AFB2 (22.6%).

As shown in Table 2, the aflatoxin concentrations varied between 0.03 μg/kg and 2.24 μg/kg, with a mean group value of 0.21 μg/kg. The highest values were reported in the *bradj* samples, with four samples ranging between 1.02 and 2.24 μg/kg. The European Union has established maximum contents of AFB1 and total aflatoxins (AFs) in commodities at high risk of contamination, such as dried figs (6 μg AFB1/kg and 10 μg/kg AFs/kg, respectively) and dates (2 μg AFB1/kg and 4 μg/kg AFs/kg, respectively). None of the analyzed *bradj* samples exceeded these maximum levels.

As shown in Table 2, 10 out of 29 (34.5%) dried fig samples were contaminated with at least one type of aflatoxin, but AFB2 was not detected in any samples. However, AFG1 was the most prevalent mycotoxin (24.1%), followed by AFB1 (17.2%) and AFG2 (10.3%), with a range of concentration between 0.02 and 0.30 μg/kg. Aflatoxins B1, G1 and G2 co-occurred in several dried fig samples, in agreement with Azaiez et al. [32] in a study on dried figs from Tunisia. The results obtained in the present study concurred with those of other studies carried out in Morocco [33] and Spain [34] that report low aflatoxin concentrations of 0.28 μg/kg and 0.62 μg/kg, respectively, in dried figs. However, in a previous study carried out in Algeria, the prevalence of AFB1 was 75.7%, and the concentration levels ranged from 5.89 to 83.4 μg/kg [6].

There are not many studies available on the presence of AFs in dates or products containing dates as an ingredient, despite being a dried fruit that is highly susceptible to these mycotoxins [35]. In the present study, the incidence of aflatoxins in dates was low (5% samples), but these mycotoxins were detected in all the analyzed samples of *bradj*, a traditional date-filled cookie, with a mean concentration of 0.87 μg/kg. Perhaps this means that the quality of the date paste used in the production of this sweet pastry should be better controlled for mycotoxins. Thus, systematic monitoring and awareness programs, especially for producers and consumers, might be a practical approach to reduce the risk. In addition, regulations should be laid down for the presence of mycotoxins in traditional food products that are widely consumed by the population.

The low incidence of AFs in dates (5%) is in line with other studies published in Egypt, with 4% incidence [36], and Pakistan, with 10% prevalence [37]. In a study carried out in Spain, AFs were not detected in dates [32]. Regarding sweet pastries containing dates, Iqbal et al. [35] reported a study in Pakistan in which 31.6% of the samples were positive for aflatoxins, with a maximum level of 16.70 μg/kg.

#### 3.1.3. Occurrences of Major Mycotoxins in Cereal-Based Products

Cereals and their derived products are widely consumed in Northern African countries. The importance of cereals is that they are the raw material for flour and semolina, which, in turn, are the main ingredients of traditional products such as couscous, *rechta* noodles and *metlou* bread. In this regard, 71 cereal products from Algerian markets were analyzed in the present study, and the results obtained are summarized in Table 3.

According to the analytical results of this group, AFs were detected in 47.9% of the samples, OTA in 16.9% and DON in 85.9%, while zearalenone was not detected in any samples. The pattern of aflatoxin contamination was very different from that observed in nuts and dried fruits, as the predominant aflatoxin in cereal-based products was AFG1 (46.5% of samples), followed at a far distance by AFB1 (1.4% of samples). The aflatoxin contamination levels were generally low: between 0.02 and 0.15 μg/kg. The mycotoxin OTA was detected in couscous, *rechta* and *metlou* samples, with a prevalence between 5.6 and 26.9%, and a mean concentration between 0.06 and 0.20 μg/kg. However, DON occurred in more than 75% of the samples of each type, with a mean concentration ranging from 90 to 123 μg/kg.

Couscous is a cereal product traditionally consumed in North African and Arab countries [7]. Although couscous is a product prepared from durum wheat semolina, it can also be made from a mixture of durum and soft wheat. This product can be homemade and traditionally prepared or manufactured in the industry. For this analyzed food, none of the couscous samples were contaminated with AFs, which differs from previous studies on wheat semolina conducted in neighboring Morocco [38], in which one couscous sample contained 31.1 μg AFB1/kg and 50.7 μg/kg of total AFs.

AFG1 was the most occurring aflatoxin found in the *rechta* (57.7%) and *metlou* samples (100%), but at low concentrations ranging between 0.02 and 0.15 μg/kg. Only two of the *metlou* and *rechta* samples presented contamination with AFG2 (0.03 μg/kg) and AFB1 (0.02 μg/kg), respectively. It is noteworthy that bread, such as *metlou*, and pasta, such as *rechta*, are made of wheat semolina and are the staple foods for the majority of the Algerian population [7]. None of the samples exceeded the maximum levels of AFs established by the EU and Algerian regulations for cereals and their derived products.

Moreover, similar results to our study were obtained in Morocco by Bouafifssa et al. [39], who reported a 1.9% incidence of AFB1 in pasta samples, with a mean of 0.25 μg/kg, while AFB2, G1 and G2 were not detected. In contrast, Hathout et al. [40] detected both AFG1 and AFG2 in 100% of wheat grain samples from Egypt. Although there are no regulations for AFG1 and AFG2 alone, the increase in the AFG1 concentrations in wheat and its derived products is considered dangerous to public health, as it follows AFB1 in toxicity [40].

The mycotoxin OTA was detected in couscous (14.8% of samples), *rechta* (26.9% of samples) and *metlou* (5.6% of samples) at levels ranging from 0.10 to 2.79 μg/kg, as shown in Table 3. Currently, no regulation for the maximum levels of OTA in foods are in force in Algeria. Compared to European legislation, no sample surpassed the maximum levels established for OTA in cereal products (3 μg/kg). In Morocco, Zinedine et al. [38] did not detect OTA in a study of cereal-based products, while Tabarini et al. [41] observed that 22% of semolina and pasta samples were contaminated with OTA at levels between 0.1 and 6.8 μg/kg. Other investigations on semolina and flours from Algeria [42] and Tunisia [43] reported elevated OTA levels of between 0.16 and 34.75 μg/kg and between 0.7 and 24.3 μg/kg, respectively.

Cereals and cereal products are a staple food in the diet of Algerians. However, the risk of contamination with mycotoxins, such as AFs and OTA, makes it necessary to consider the potential health risks. Badji et al. [44] tested a detoxification procedure for AFB1 and OTA based on lactic acid bacteria (LAB) strains isolated from Algerian fermented foods. The LAB strains tested reduced both mycotoxins in vitro, suggesting that they could be used as manufacturing aids to reduce the mycotoxin levels in bread.

As expected, cereal products were contaminated by the deoxynivalenol (DON) mycotoxin. The total incidence in couscous, *rechta* and *metlou* varied between 77.8% and 96.2%, and the range was between 33 μg/kg and 470 μg/kg. Compared to the European legislation, no sample exceeded the maximum levels set for DON in cereal products, such as pasta (750 μg/kg) and bread (500 μg/kg). The DON concentrations found in this survey are in accordance with those reported in Morocco by Zinedine et al. [38], which ranged from 20.6 to 106.6 μg/kg in couscous samples, and in Italy by Tolosa et al. [45], who found DON in pasta products at 20.9 μg/kg–247.3 μg/kg. These results were also similar to studies conducted by Mahdjoubi et al. [16] in Algeria, with a mean content in wheat of 223 μg DON/kg, and González-Osnaya et al. [46] in Spain, who detected DON at 42.5 μg/kg in bread and at 137.1 μg/kg in pasta.

### 3.2. Exposure Assessment and Risk Characterization

The risk exposure of the adult population in Algeria to mycotoxins through the consumption of nuts, dried fruits and cereal-based products was determined. The estimated daily intakes (EDIs) were obtained for every analyzed mycotoxin as indicated in the following formula:

EDI = (K [g/day] × Cm [μg/kg])/bw [kg],(1)

where EDI is the estimated daily intake for each mycotoxin; K is the food intake (g/day); Cm is the mean concentration of each mycotoxin (μg/kg); Bw is the body weight for adults (70 kg). The food intake was based on the individual consumption of peanuts (2 g/day), tree nuts (4 g/day), dried figs (10 g/day), dates (60 g/day), *bradj* (serving of 60 g) and cereal products (a serving of 100 g of couscous, *rechta* noodles or *metlou* bread). These food supply quantities were based on the Algerian Food Balance for 2019 [31] and a questionnaire survey carried out during the sampling year 2019. The mean concentration of mycotoxins was calculated by assigning a value of LD/2 to the left-censored (LC) data [47,48].

AFB1 is both genotoxic and carcinogenic [11], while the genotoxic potential of OTA is unclear [12]. Nevertheless, the margin of exposure (MOE) was applied to identify the risk characterization of these mycotoxins in the present study. An MOE value of 10,000 (ten thousand) or higher is considered of low concern from a public health point of view for genotoxic and carcinogenic substances [49]. For AFs, MOE values were calculated by dividing the reference benchmark dose lower confidence level BMDL_10_ of 0.4 μg/kg bw/day by the exposure estimates (EDIs) [11]. In this study, the same value of the BMDL was used for the MOE calculations of AFB2, G1 and G2. Thus, MOE values, according to this approach, serve to prioritize the need for risk management measures [50]. For OTA, the MOE values were calculated by dividing the reference BMDL_10_ of 14.5 μg/kg bw/day by the exposure estimates (EDIs) [12].

The AF and OTA results presented in Table 4 allowed for an evaluation of the risk characterization based on the exposure expressed in estimated daily intakes and MOE values. In this table, we highlight in bold style the MOE values that are lower than 10,000 (ten thousand) because they indicate a health risk. The higher estimated daily exposure for AFB1 was determined in *bradj* at 0.617 ng/kg bw/day, allowing for the calculation of a margin of exposure (MOE) value of 648, which raises a public health concern for Algerian consumers. Traditional foods such as *bradj*, *rechta* and *metlou* were also sources of the aflatoxins B2 and G1, with MOE values ranging from 4000 to 9333, indicating a potential risk for the Algerian population. An MOE of this magnitude should drive the implementation of risk management measures to reduce human exposure.

However, the aflatoxin MOE values for peanuts, almonds, walnuts, dried figs, dates and couscous were all greater than ten thousand. In general, MOE values of 10,000 or higher would be of low concern from a public health point of view and might be considered as a low priority for risk management actions.

The risk posed by the dietary exposure to AFs through the consumption of nuts, dried fruits and cereal products has been reviewed worldwide [51]. The results obtained in this study are similar to the AFB1 exposure values reported in several publications. For example, Kortei et al. [52] reported EDIs of 0.07 ng/kg bw/day for peanuts in Ghana, 0.02 ng/kg bw/day for dried figs in Turkey [53], 0.04 ng/kg bw/day for dried figs in Iran [47] and 0.08 ng/kg bw/day for cereal products in Turkey [50]. In contrast, other researchers, such as Kooprasetying et al. [54] in Thailand and Alim et al. [55] in Pakistan, conducted AFB1 exposure assessments, and the estimated daily intake values for peanut (0.60 ng/kg bw/day) and noodle (0.59 ng/kg bw/day) consumption were higher than those found in the present work.

The OTA MOE values for nuts, dried fruits and cereal-based products were higher than 10,000, indicating low concern from a public health standpoint. Thus, the mean exposure to OTA across cereal-based products reached values from 0.086 to 0.286 ng/kg bw/day, corresponding to MOE values well above the 10,000-threshold value.

Considering the mean exposure, the estimated values were lower than the EDIs through the consumption of cereal products in France (0.28–1.91 ng/kg bw/day), Ireland (0.47–3.11 ng/kg bw/day) and Morocco (4 ng/kg bw/day) reported by Sirot et al. [56], FSAI [57] and Zinedine et al. [38], respectively.

The IARC has classified DON as Group 3 [58], and a tolerable daily intake (TDI) of 1 μg/kg bw/day has been established [13]. Once the EDI was calculated for DON (μg DON/kg bw/day), the risk was estimated as the percentage of the tolerable daily intake (%TDI), calculated as the ratio of the EDI to the TDI (μg/kg bw/day), as follows:

%TDI = (EDI/TDI) × 100,(2)

The estimated daily intakes for DON through the consumption of cereal-based products in the Algerian population were 0.167, 0.176 and 0.127 μg DON/kg bw/day for couscous, *rechta* noodles and *metlou* bread, respectively, the intakes of which were 16.7, 17.6 and 12.7% of the TDI, respectively. Similarly, an Italian study evaluating DON exposure from pasta consumption found an estimated intake of about 15% of the TDI [59]. In previous studies, dietary exposure values through the consumption of wheat products were estimated to be 0.85 and 0.79 μg DON/kg bw/day for the Algerian [16] and Brazilian [60] populations, respectively.

## 4. Conclusions

This paper provides information related to the occurrences, exposure assessment and risk characterization of aflatoxins, ochratoxin A and deoxynivalenol in staple and traditional foods from Algeria. Aflatoxins were found in 37.9% of the analyzed food products, and 2% of the samples (corresponding to peanuts) exceeded the EU maximum level for AFB1 (2 μg/kg). Likewise, all the *bradj* and *metlou* samples were contaminated with aflatoxins, which poses a potential health risk because these traditional products are widely consumed by the Algerian population. The aflatoxin exposure of average Algerian consumers may represent a safety concern, as the estimated intakes from the consumption of *bradj*, *rechta* and *metlou* indicated margin of exposure (MOE) values that are likely to be harmful.

The mycotoxin OTA was only present in cereal-based products (16.9% occurrence), and, based on the levels found, the estimated intake does not represent a health concern, as they were all within the margin of exposure (MOE values above 10,000). Finally, it should be noted that the assessment of the dietary exposure to DON from the consumption of cereal-based foods reached estimated intakes ranging from 12.7% to 17.6% of the TDI values.

Algerian producers and regulators may not be aware of the mycotoxin problem in commodities due to the lack of a national strategy to study and control these contaminants along the food chain, as well as the absence of maximum mycotoxin limits in many products. Thus, these results may help to establish mycotoxin control measures and future directions for mycotoxin research for the mitigation of the health risks to humans.

## Figures and Tables

**Table 1:** Occurrences and levels of aflatoxins (B1, B2, G1, G2 and sum of aflatoxins) and ochratoxin A (OTA) in nuts. Results expressed as μg/kg.

Food	Descriptive	AFB1	AFB2	AFG1	AFG2	Sum AFs	OTA
Peanuts (n = 24)	% Positive	62.5%	62.5%	45.8%	29.2%	62.5%	0.0%
	Mean ± SD	0.92 ± 1.34	0.16 ± 0.23	0.17 ± 0.37	0.08 ± 0.19	1.31 ± 1.89	<LD ^1^
	Maximum	5.25	0.83	1.42	0.74	6.21	--
Almonds (n = 21)	% Positive	9.5%	9.5%	0.0%	0.0%	9.5%	0.0%
	Mean ± SD	0.01 ± 0.01	0.01 ± 0.01	<LD	<LD	0.02 ± 0.02	<LD
	Maximum	0.05	0.02	--	--	0.07	--
Walnuts (n = 20)	% Positive	0.0%	0.0%	0.0%	0.0%	0.0%	0.0%
	Mean ± SD	<LD	<LD	<LD	<LD	<LD	<LD
	Maximum	--	--	--	--	--	--
Total nuts (n = 65)	% Positive	26.2%	26.2%	16.9%	10.8%	26.2%	0.0%
	Mean ± SD	0.35 ± 0.92	0.06 ± 0.16	0.07 ± 0.24	0.04 ± 0.12	0.49 ± 1.30	<LD

^1^ LD: limit of detection of 0.02 μg/kg for each of the aflatoxins and 0.10 μg/kg for ochratoxin A.

**Table 2:** Occurrences and levels of aflatoxins (B1, B2, G1, G2 and sum of aflatoxins) and ochratoxin A (OTA) in dried fruits. Results expressed as μg/kg.

Food	Descriptive	AFB1	AFB2	AFG1	AFG2	Sum AFs	OTA
Dried figs (n = 29)	% Positive	17.2%	0.0%	24.1%	10.3%	34.5%	0.0%
	Mean ± SD	0.01 ± 0.01	<LD ^1^	0.03 ± 0.06	0.02 ± 0.03	0.04 ± 0.07	<LD
	Maximum	0.05	--	0.30	0.17	0.32	--
Dates (n = 20)	% Positive	5.0%	5.0%	0.0%	0.0%	5.0%	0.0%
	Mean ± SD	0.02 ± 0.05	0.01 ± 0.02	<LD	<LD	0.03 ± 0.07	<LD
	Maximum	0.23	0.10	--	--	0.33	--
*Bradj* pastries (n = 13)	% Positive	100.0%	100.0%	100.0%	0.0%	100.0%	0.0%
	Mean ± SD	0.72 ± 0.58	0.09 ± 0.06	0.06 ± 0.01	<LD	0.87 ± 0.64	<LD
	Maximum	1.97	0.20	0.07	--	2.24	--
Total dried fruits (n = 62)	% Positive	30.6%	22.6%	32.3%	4.8%	38.7%	0.0%
	Mean ± SD	0.16 ± 0.39	0.03 ± 0.04	0.03 ± 0.04	0.01 ± 0.02	0.21 ± 0.45	<LD

^1^ LD: limit of detection of 0.02 μg/kg for each of the aflatoxins and 0.10 μg/kg for ochratoxin A.

**Table 3:** Occurrences and levels of aflatoxins (B1, B2, G1, G2 and sum of aflatoxins), ochratoxin A (OTA) and deoxynivalenol (DON) in cereal-based products. Results expressed as μg/kg.

Food	Descriptive	AFB1	AFB2	AFG1	AFG2	Sum AFs	OTA	DON
Couscous (n = 27)	% Positive	0.0%	0.0%	0.0%	0.0%	0.0%	14.8%	77.8%
	Mean ± SD	<LD ^1^	<LD	<LD	<LD	<LD	0.20 ± 0.56	117 ± 97
	Maximum	--	--	--	--	--	2.79	470
*Rechta* noodles (n = 26)	% Positive	3.8%	0.0%	57.7%	0.0%	61.5%	26.9%	96.2%
	Mean ± SD	0.01 ± 0.01	<LD	0.03 ± 0.03	<LD	0.03 ± 0.03	0.17 ± 0.44	123 ± 63
	Maximum	0.02	--	0.09	<LD	0.09	2.28	280
*Metlou* bread (n = 18)	% Positive	0.0%	0.0%	100.0%	5.6%	100.0%	5.6%	83.3%
	Mean ± SD	<LD	<LD	0.07 ± 0.03	0.01 ± 0.01	0.07 ± 0.03	0.06 ± 0.02	90 ± 60
	Maximum	--	--	0.15	0.03	0.15	0.15	190
Total cereal-based (n = 71)	% Positive	1.4%	0.0%	46.5%	1.4%	47.9%	16.9%	85.9%
	Mean ± SD	0.01 ± 0.01	<LD	0.03 ± 0.03	0.01 ± 0.01	0.03 ± 0.03	0.15 ± 0.43	112 ± 77

^1^ LD: limit of detection of 0.02 μg/kg for each of the aflatoxins, 0.10 μg/kg for ochratoxin A and 33 μg/kg for deoxynivalenol.

**Table 4:** Estimated daily intake (EDI) (as ng/kg bw/day) and margin of exposure (MOE) values of aflatoxins B1, B2, G1 and G2 and ochratoxin A (OTA) from analyzed foods.

Food	Parameter	AFB1	AFB2	AFG1	AFG2	OTA
Peanuts	EDI	0.026	0.005	0.005	0.002	LC ^1^
	MOE	15,217	87,500	82,353	175,000	-
Almonds	EDI	0.001	0.001	LC	LC	LC
	MOE	560,000	560,000	-	-	-
Walnuts	EDI	LC	LC	LC	LC	LC
	MOE	-	-	-	-	-
Dried figs	EDI	0.001	LC	0.004	0.003	LC
	MOE	280,000	-	93,333	140,000	-
Dates	EDI	0.017	0.009	LC	LC	LC
	MOE	23,333	46,667	-	-	-
*Bradj* pastries	EDI	0.617	0.076	0.051	LC	LC
	MOE	**648** ^2^	**5185**	**7778**	-	-
Couscous	EDI	LC	LC	LC	LC	0.286
	MOE	-	-	-	-	50,750
*Rechta* noodles	EDI	0.014	LC	0.043	LC	0.243
	MOE	28,000	-	**9333**	-	59,706
*Metlou* bread	EDI	LC	LC	0.100	0.014	0.086
	MOE	-	-	**4000**	28,000	169,167

^1^ LC: left-censored data; ^2^ highlighted in bold style are the MOE values that are lower than 10,000 (ten thousand), indicating health risk.

## Data Availability

The data presented in this study are available in the article.

## Supplementary Materials

The following supporting information can be downloaded at https://www.mdpi.com/article/10.3390/toxins15070449/s1, Figure S1. Calibration curves for the four aflatoxins (0.15 to 1 ng/mL); Figure S2. Calibration curves for aflatoxin B1 (1.5 to 5 ng/mL), OTA (0.25 to 1.5 ng/mL) and DON (50 to 500 ng/mL).; Figure S3. LC chromatogram of a peanut sample contaminated with the four aflatoxins; Figure S4. LC chromatogram of a peanut sample contaminated with the four aflatoxins; Figure S5. LC chromatogram of a fig sample contaminated with aflatoxins G1 and B1; Figure S6. LC chromatogram of a date sample contaminated with aflatoxins B1 and B2; Figure S7. LC chromatogram of a couscous sample contaminated with deoxynivalenol; Figure S8. LC chromatogram of a couscous sample contaminated with ochratoxin A.
