# Supplementary material for: Occurrence and Exposure Assessment of Major Mycotoxins in Foodstuffs from Algeria
Source: Toxins (Basel). 2023 Jul 6;15(7):449. doi: 10.3390/toxins15070449 (PMC10467145; doi:10.3390/toxins15070449)
Supplement: Supplementary file 1 [file toxins-15-00449-s001.zip › toxins-2460001-supplementary.pdf]

Supplementary Materials: Occurrence and exposure assessment of major mycotoxins in foodstuffs from Algeria

Azem Belasli, Marta Herrera, Agustín Ariño and Djamel Djenane

Toxins

Aflatoxin B1	
ng/mL	Area
0.15	144.832
0.3	263.934
0.5	435.253
0.75	671.099
1	883.400

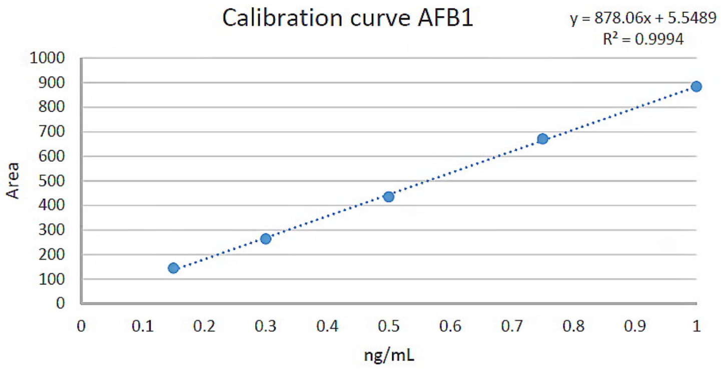

Aflatoxin B2	
ng/mL	Area
0.15	81.799
0.3	147.484
0.5	243.685
0.75	361.154
1	463.876

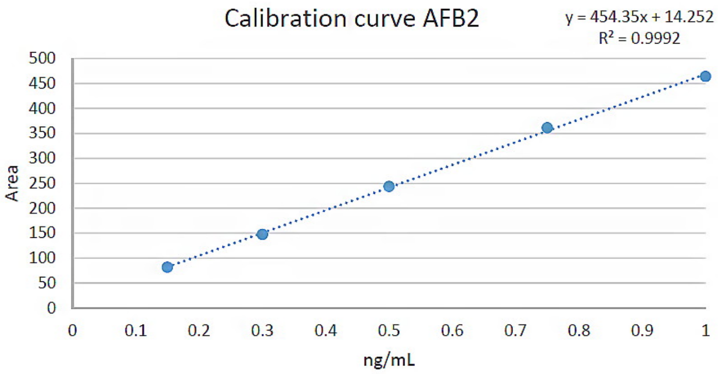

Aflatoxin G1	
ng/mL	Area
0.15	84.742
0.3	159.112
0.5	252.451
0.75	373.698
1.5	766.404

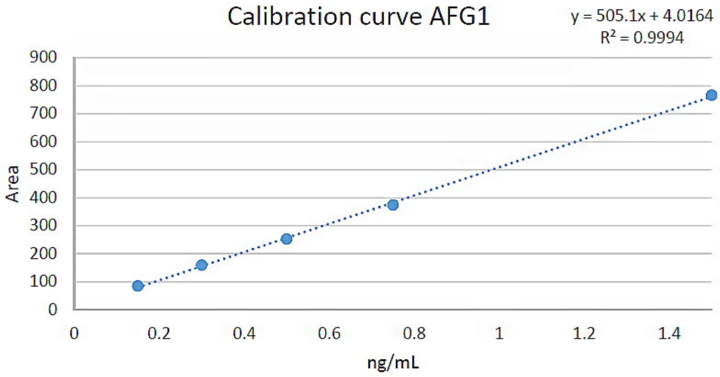

Aflatoxin G2	
ng/mL	Area
0.15	30.077
0.3	72.824
0.5	116.332
0.75	173.47
1	233.307

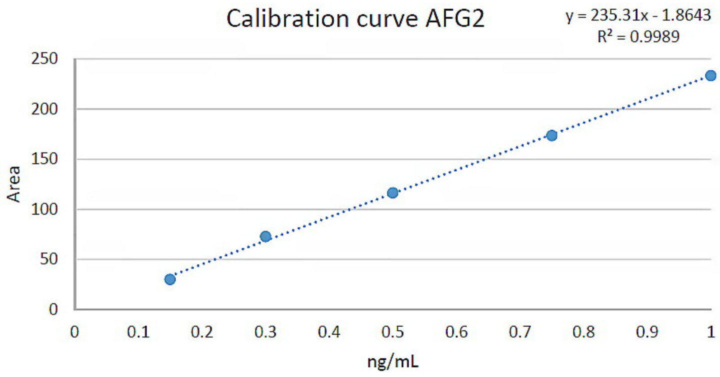

Figure S1. Calibration curves for the four aflatoxins (0.15 to 1 ng/mL).

Aflatoxin B1	
ng/mL	Area
1.5	1320.335
2.5	2120.769
3	2543.552
3.5	3076.064
5	4400.330

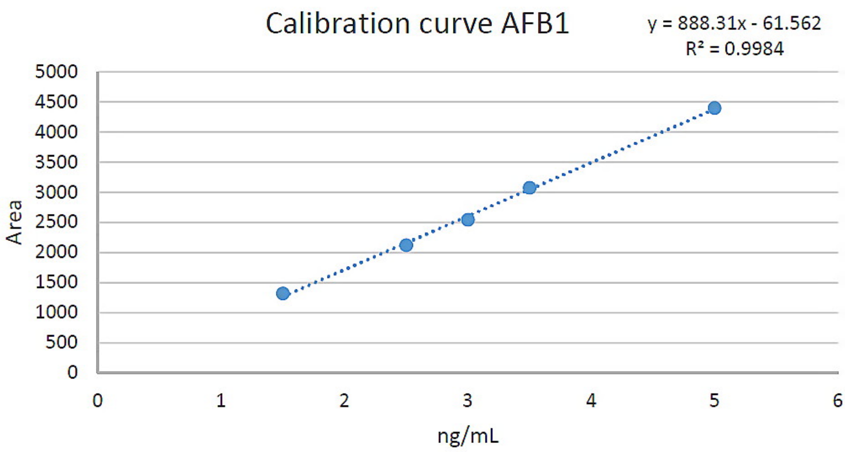

Ochratoxin A (OTA)	
ng/mL	Area
0.25	1.3209
0.5	2.04381
0.75	3.04529
1	4.3254
1.5	6.588

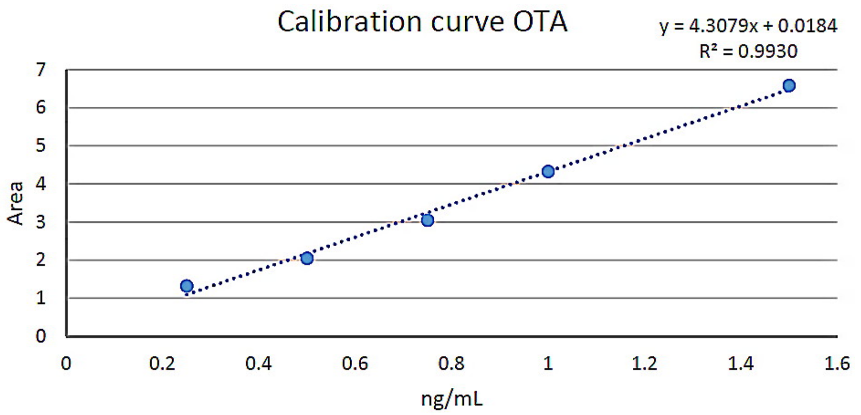

Deoxynivalenol (DON)	
ng/mL	Area
50	10.56
75	16.36
100	18.49
250	37.6
500	65.18

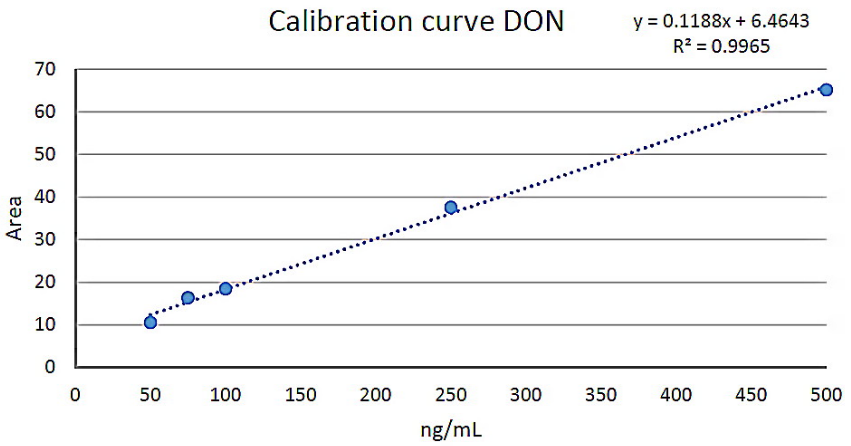

Figure S2. Calibration curves for aflatoxin B1 (1.5 to 5 ng/mL), OTA (0.25 to 1.5 ng/mL) and DON (50 to 500 ng/mL).

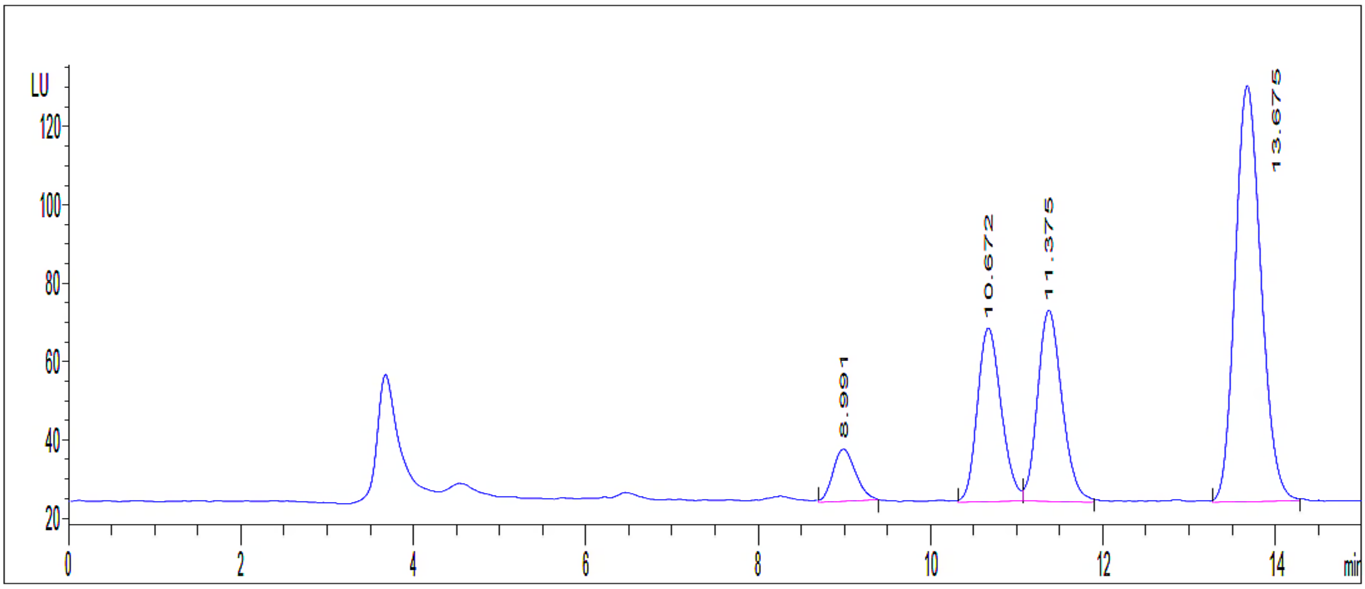

Figure S3. LC chromatogram of a peanut sample contaminated with the four aflatoxins.

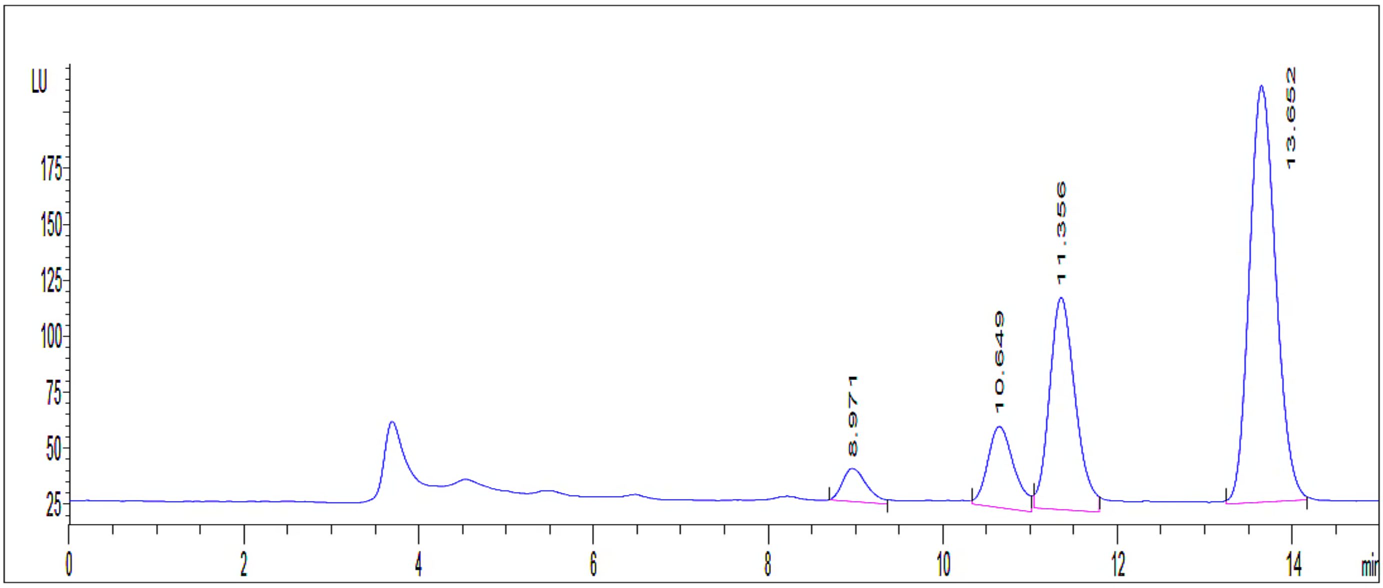

Figure S4. LC chromatogram of a peanut sample contaminated with the four aflatoxins.

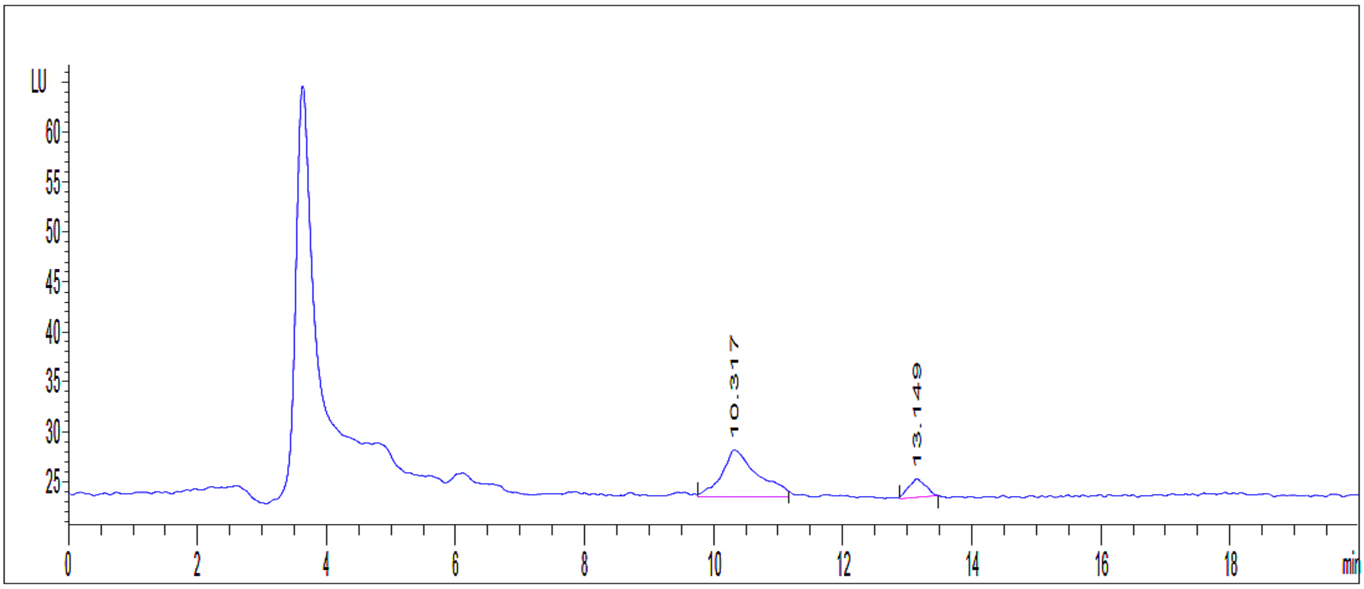

Figure S5. LC chromatogram of a fig sample contaminated with aflatoxins G1 and B1.

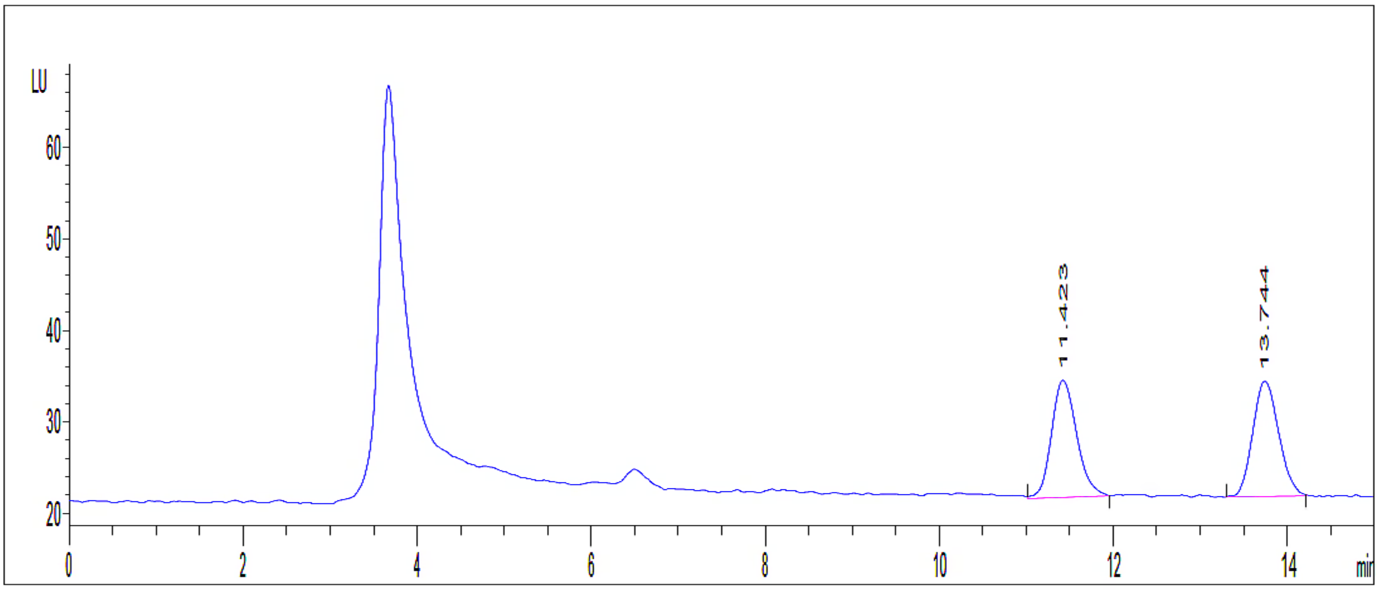

Figure S6. LC chromatogram of a date sample contaminated with aflatoxins B1 and B2.

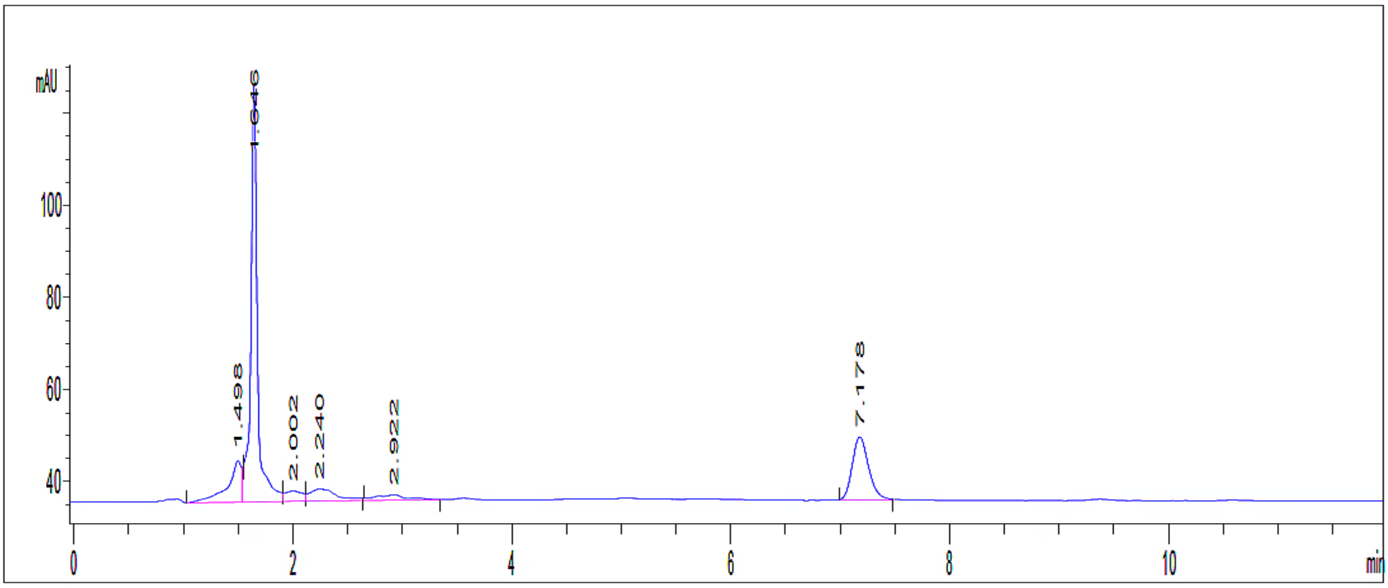

Figure S7. LC chromatogram of a couscous sample contaminated with deoxynivalenol.

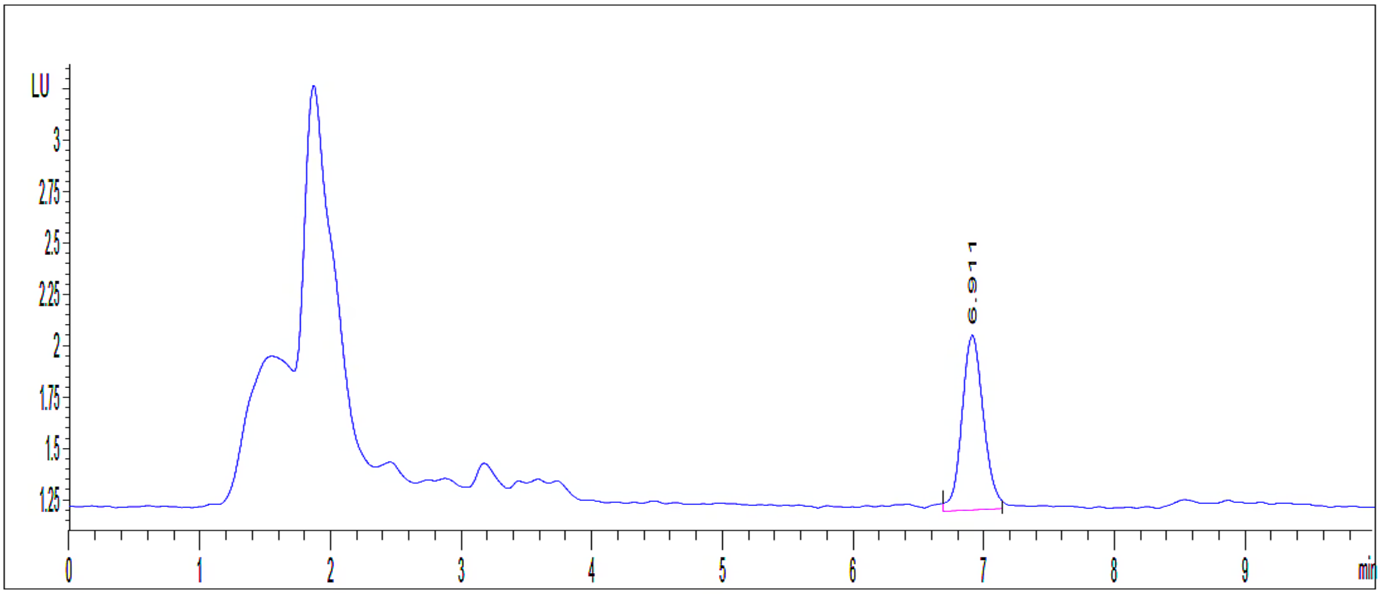

Figure S8. LC chromatogram of a couscous sample contaminated with ochratoxin A.
